# Extruded Pseudocereal Snacks Mathematical Modelling Approaches for Prediction and Optimisation: A Review

**DOI:** 10.3390/foods15162854

**Published:** 2026-08-15

**Authors:** Biljana Lončar, Miloš Radosavljević, Jelena Filipović, Ivica Djalović, Milenko Košutić, Vladimir Filipović, Milica Nićetin

**Affiliations:** 1 Faculty of Technology Novi Sad, University of Novi Sad, Bulevar Cara Lazara 1, 21000 Novi Sad, Serbia; biljanacurcicc@gmail.com (B.L.); vladaf@uns.ac.rs (V.F.); milican@uns.ac.rs (M.N.); 2 Institute of Food Technology, University of Novi Sad, Bulevar Cara Lazara 1, 21000 Novi Sad, Serbia; jelena.filipovic@fins.uns.ac.rs (J.F.); milenko.kosutic@fins.uns.ac.rs (M.K.); 3 Institute of Field and Vegetable Crops, National Institute of the Republic of Serbia, Maxim Gorki 30, 21000 Novi Sad, Serbia; ivica.djalovic@ifvcns.ns.ac.rs

**Keywords:** extrusion, mathematical modelling, optimisation, response surface methodology, artificial neural networks, snack foods, quinoa, amaranth, buckwheat, machine learning

## Abstract

Pseudocereals such as quinoa, amaranth, and buckwheat have attracted increasing attention as ingredients for extruded snack products because of their nutritional value, gluten-free status, and content of bioactive compounds. The quality of extruded products is governed by complex interactions among processing variables, including barrel temperature, screw speed, feed moisture content, and formulation characteristics. As a result, mathematical modelling has become an important tool for predicting product properties and identifying suitable processing conditions. This review summarizes modelling approaches applied to extruded food products with a focus on pseudocereal extrusion. Particular emphasis is placed on response surface methodology (RSM), artificial neural networks (ANNs), adaptive neuro-fuzzy inference systems (ANFIS), support vector regression (SVR), and hybrid optimisation strategies. Published studies indicate that RSM remains the most commonly used approach because of its simplicity and interpretability, while ANN-based models generally provide much higher predictive accuracy when strong nonlinear relationships are present. The widespread use of small experimental datasets and limited external validation remains a major challenge for the practical implementation of advanced machine-learning models. This review examines the strengths and limitations of current modelling approaches and discusses future opportunities for integrating predictive models with digital manufacturing frameworks.

## 1. Introduction

Interest in pseudocereals has greatly increased during the last two decades, mainly because of the growing demand for gluten-free and nutritionally enhanced foods. Quinoa (*Chenopodium quinoa* Willd.), amaranth (*Amaranthus* spp.), and buckwheat (*Fagopyrum* spp.) are among the studied pseudocereal species due to their favourable nutritional composition and suitability for incorporation into a variety of food products [1,2].

Unlike typical cereals, pseudocereals contain proteins with a more balanced essential amino acid profile and are important sources of dietary fibre, minerals, vitamins, and phytochemicals [3,4]. The use of pseudocereals in bakery products, breakfast cereals, pasta, and snack foods has expanded in response to consumer interest in foods that combine nutritional quality with specific benefits for health [5,6]. Quinoa is particularly valued for its protein quality and mineral content, amaranth for its squalene-rich lipid fraction, and buckwheat for its flavonoids, especially rutin [7,8,9,10].

Their distinct starch, protein, and fibre characteristics influence processing behaviour and product quality differently from traditional cereal systems. Understanding these effects remains important for the development of acceptable and nutritionally valuable pseudocereal-based foods [11,12,13,14].

Extrusion cooking is one of the most widely used technologies for producing expanded snack foods because it combines mixing, cooking, shaping, and texturisation into a single continuous process. During extrusion, raw materials are subjected to elevated temperatures (100–200 °C), mechanical shear, and pressure (2–20 MPa), causing extensive physicochemical transformations that determine the structure and quality of the final product [15,16].

The versatility of extrusion technology allows manufacturers to process a wide range of formulations while controlling product characteristics by adjustment of process variables such as barrel temperature, screw speed, feed moisture content, feed rate, and die configuration [17,18,19]. These variables rarely act independently. Instead, their effects are highly interactive, often producing nonlinear responses in expansion, texture, density, colour, and nutritional properties [20].

For pseudocereal-based products, process optimisation is particularly challenging because their compositional characteristics influence starch gelatinisation, protein denaturation, melt rheology, and moisture distribution differently than conventional cereal systems. As a result, small changes in operating conditions may lead to substantial differences in product quality [21,22].

The development of high-quality extruded pseudocereal snacks requires balancing multiple technological and nutritional objectives simultaneously [23]. From a technological perspective, sufficient expansion and desirable texture are essential for consumer acceptance [24,25]. The high protein and fibre contents characteristic of pseudocereals may restrict bubble growth during die expansion, resulting in denser products with reduced crispness [26].

Nutritional considerations add further complexity. Although extrusion can improve digestibility and reduce certain antinutritional compounds, it may also affect thermally sensitive bioactive constituents such as rutin, tocopherols, polyphenols, and squalene [27]. The extent of these changes depends on both formulation and processing conditions. Optimisation strategies must account not only for physical quality attributes but also for nutritional and functional characteristics [28,29,30].

These interacting phenomena make extrusion systems difficult to describe using simple linear relationships. Traditional trial-and-error approaches are often insufficient to identify optimal operating conditions, particularly when multiple responses must be considered simultaneously [31].

### 1.1. Scope and Objectives of the Review

The objective of this review is to critically summarize and compare mathematical modelling approaches used to predict and optimise the quality of extruded pseudocereal products. Particular attention is given to quinoa, amaranth, and buckwheat because these species represent the most widely investigated pseudocereals in extrusion research.

The central hypothesis of this review is that the complex and highly nonlinear relationships between extrusion conditions, pseudocereal composition, and product quality are unlikely to be adequately described by a single modelling framework. Rather, the suitability and predictive performance of a modelling approach depend on the characteristics of the extrusion system, dataset size, response complexity, validation strategy, and intended application.

The specific objectives of this review are to:

(1) summarize the compositional characteristics and extrusion behaviour of quinoa, amaranth, buckwheat, and their composite formulations (Section 2);

(2) identify the principal physical, functional, nutritional, and sensory quality attributes used as responses in extrusion modelling studies (Section 3);

(3) critically examine and compare the major mathematical modelling approaches applied to extrusion, including RSM, ANNs, hybrid machine-learning methods, and mechanistic models (Section 4);

(4) evaluate the relationships between extrusion process variables, product quality responses, model performance, and optimisation strategies (Section 5 and Section 6); and

(5) identify current methodological limitations, emerging modelling trends, and future research priorities for data-driven pseudocereal extrusion processing (Section 7 and Section 8).

Although several review papers have discussed extrusion processing of pseudocereals or mathematical modelling in food processing separately, to the best of our knowledge, no previous review has comprehensively integrated these topics by critically comparing conventional statistical approaches with modern machine-learning techniques specifically for pseudocereal extrusion systems. Previous reviews have mainly focused on processing conditions, nutritional quality, or product development, whereas limited attention has been paid to comparing model performance, data requirements, validation strategies, and practical applicability across different modelling frameworks.

Unlike previous review articles, this review provides an integrated perspective on predictive modelling and optimisation in pseudocereal extrusion, identifies current methodological limitations, and outlines future research directions toward data-driven and intelligent extrusion process design.

### 1.2. Literature Search Strategy and Review Methodology

To ensure broad coverage of the available literature, a structured literature search was conducted in the Scopus, Web of Science Core Collection, ScienceDirect, and Google Scholar databases. The search covered publications from January 2000 to May 2026.

The search strategy was based on combinations of the following keywords using Boolean operators (AND/OR): (“pseudocereal”, OR “quinoa”, OR “amaranth”, OR “buckwheat”), AND (“extrusion”, OR “extrusion cooking”), AND (“response surface methodology”, OR “RSM”, OR “artificial neural network”, OR “ANN”, OR “machine learning”, OR “optimisation”, OR “ANFIS”, OR “support vector regression”, OR “genetic algorithm”).

The inclusion criteria comprised peer-reviewed articles published in English that investigated extrusion processing of pseudocereals and reported mathematical modelling, optimisation, or predictive approaches. Studies focusing on quinoa, amaranth, buckwheat, or their blends were prioritised. Representative studies involving cereal- and legume-based extrusion systems were also included when they provided methodological developments or modelling approaches relevant to pseudocereal extrusion.

The exclusion criteria included conference abstracts, patents, book chapters, non-English publications, duplicate records, and studies unrelated to extrusion processing or mathematical modelling.

The combined database searches yielded more than 700 potentially relevant database records before duplicate removal. Following duplicate removal, title and abstract screening, and full-text assessment based on the predefined inclusion and exclusion criteria, representative peer-reviewed studies most relevant to the objectives of this review were selected for qualitative synthesis. Particular attention was given to studies reporting quantitative relationships between extrusion process variables and product quality attributes, including expansion ratio, bulk density, texture, colour, water absorption index, water solubility index, nutritional properties, and bioactive compound retention.

Because the objective of this review was to provide a critical narrative synthesis rather than a formal systematic review or meta-analysis, a complete PRISMA protocol was not applied. Nevertheless, the literature search and selection process followed a structured and transparent approach based on predefined search terms, inclusion and exclusion criteria, database screening, and critical evaluation of eligible full-text articles.

The selected studies were subsequently classified into four major categories: response surface methodology and experimental design approaches, artificial neural network modelling, hybrid and machine-learning-based optimisation methods, and mechanistic and transport-based modelling approaches. This classification framework facilitated a systematic comparison of model structures, predictive capabilities, data requirements, and practical applicability in pseudocereal extrusion systems.

## 2. Pseudocereals: Properties and Extrusion Behaviour

### 2.1. Quinoa (Chenopodium quinoa Willd.)

Quinoa grain presents a complex multi-component matrix that influences its behaviour under extrusion conditions. The protein content of quinoa varieties ranges from 12 to 23% on a dry weight basis, with the major storage proteins being glutelins (37%) and prolamins (35%), supplemented by albumins and globulins; this protein fraction is distinguished by an essential amino acid score approaching or exceeding 1.0, particularly for lysine (5.1 g/100 g protein) [5,32]. The starch fraction, comprising 48–69% of dry weight, consists of approximately 11% amylose and 89% amylopectin with extremely small granule dimensions (0.4–2.0 μm), conferring rapid gelatinisation kinetics and a gelatinisation temperature range of 57–64 °C [33].

To map the evolution of pseudocereal research in food engineering, a multi-stage bibliometric analysis was conducted using the Scopus, Web of Science, ScienceDirect and Google Scholar databases (2000–2026), Figure 1, Figure 2 and Figure 3. While the initial search strings for quinoa, amaranth, and buckwheat yielded varying raw document counts—312, 245, and 198 respectively—the qualitative synthesis focused on high-impact studies utilizing advanced mathematical modelling.

**Figure 1:**
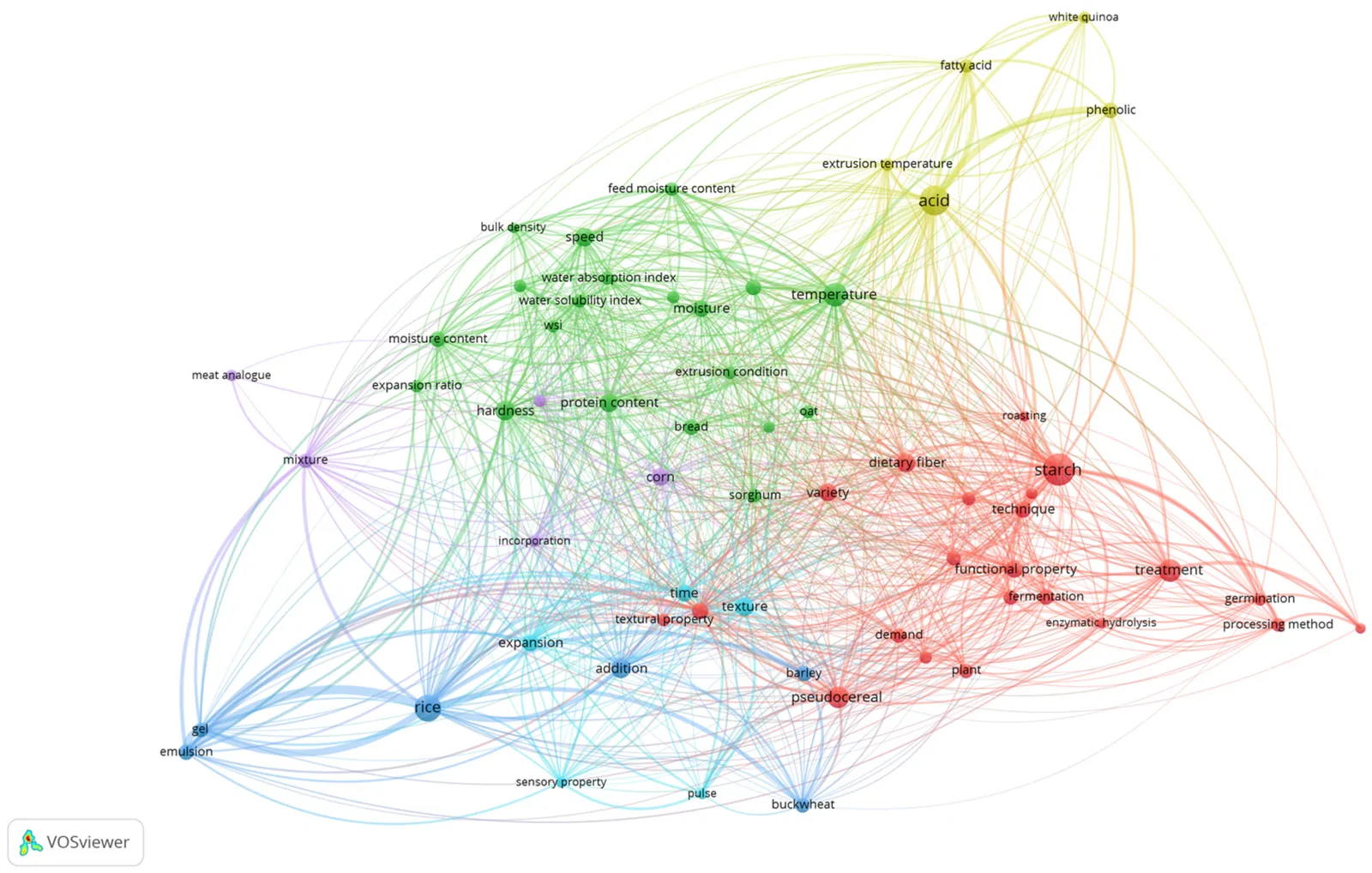
Keyword co-occurrence network map of the scientific literature on extruded pseudocereal snacks with quinoa, generated with VOSviewer 1.6.20. based on a database query (2000–2026; n = 312 documents; minimum co-occurrence threshold = 5). Node size reflects co-occurrence frequency and edge thickness denotes co-occurrence strength between paired terms.

The keyword co-occurrence network for quinoa-related extrusion research is characterized by terms associated with extrusion processing, starch functionality, nutritional properties, and product quality (Figure 1). Keywords including optimisation and modelling are also present within the network, indicating interest in predictive approaches for process and product development. The map suggests that current quinoa extrusion research is increasingly combining product quality evaluation with process optimisation methodologies.

The extrusion behaviour of quinoa is strongly influenced by interactions among temperature, moisture content, screw speed, and formulation composition. Published studies show that moderate moisture contents combined with elevated barrel temperatures favour expansion, whereas increasing proportions of quinoa protein generally reduce expansion and increase product density. These effects are commonly attributed to interactions between protein-rich matrices and starch expansion mechanisms. Besides physical changes, extrusion may improve protein digestibility and reduce antinutritional factors, although partial losses of phenolic compounds and antioxidant activity have also been reported [34,35,36,37].

### 2.2. Amaranth (Amaranthus spp.)

Amaranth grain, encompassing commercially relevant species (including *Amaranthus hypochondriacus*, *A. cruentus*, *A. caudatus*, and *A. hybridus*), is characterized by a relatively high protein content, a lipid fraction rich in squalene, and small starch granules that respond rapidly to thermomechanical treatment. These characteristics influence both processing behaviour and final product quality during extrusion [7,38,39,40].

**Figure 2:**
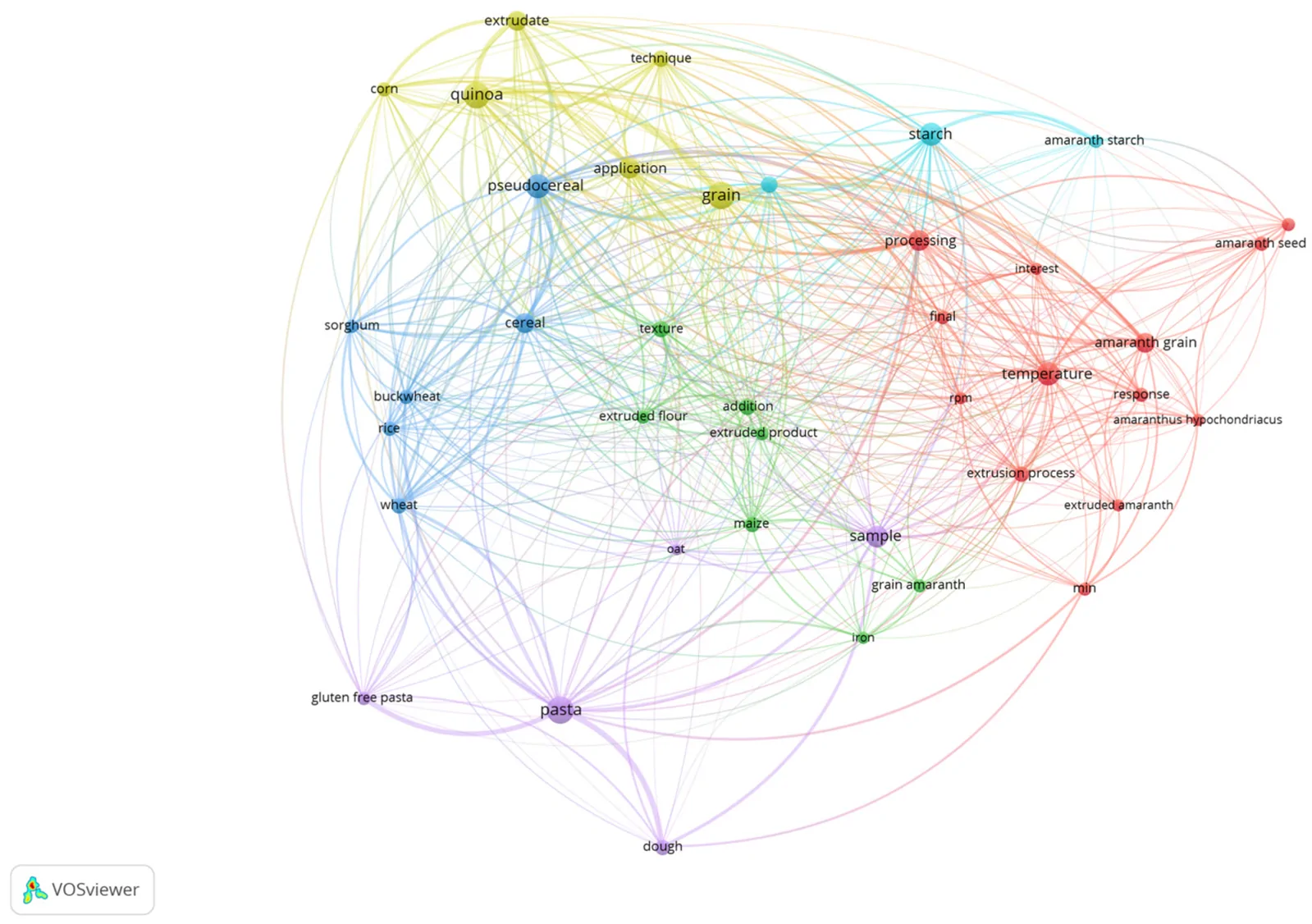
Keyword co-occurrence network map of the scientific literature on amaranth in the context of extrusion and food processing, generated with VOSviewer based on a database query (2000–2026; n = 245, minimum co-occurrence threshold = 5).

Figure 2 shows the keyword clusters identified in the amaranth extrusion literature. Frequently occurring terms are associated with extrusion processing, functional properties, antioxidant activity, and nutritional quality. The presence of optimisation-related keywords reflects increasing use of statistical and computational approaches for evaluating the effects of processing variables on product characteristics. These trends indicate that research has progressively expanded from product development toward process optimisation and quality prediction.

Published studies indicate that extrusion conditions significantly affect expansion, water absorption, digestibility, and retention of bioactive compounds, highlighting the need for optimisation strategies capable of balancing technological and nutritional objectives [38,39,40,41,42,43].

### 2.3. Buckwheat (Fagopyrum esculentum and F. tataricum)

Buckwheat occupies a unique position among pseudocereals by virtue of its exceptional concentration of rutin (quercetin-3-*O*-rutinoside), with Tartary buckwheat (*F. tataricum*) containing 0.8–2.0 g rutin per 100 g groat flour compared to 0.01–0.04 g/100 g in common buckwheat (*F. esculentum*) [9,44]. The protein content of buckwheat (11–15% dry weight) includes an essential amino acid score of 0.80–0.92, and the starch fraction (56–70%) exhibits a gelatinisation temperature range of 58–68 °C with a higher proportion of slowly digestible and resistant starch compared to most cereals [45,46].

**Figure 3:**
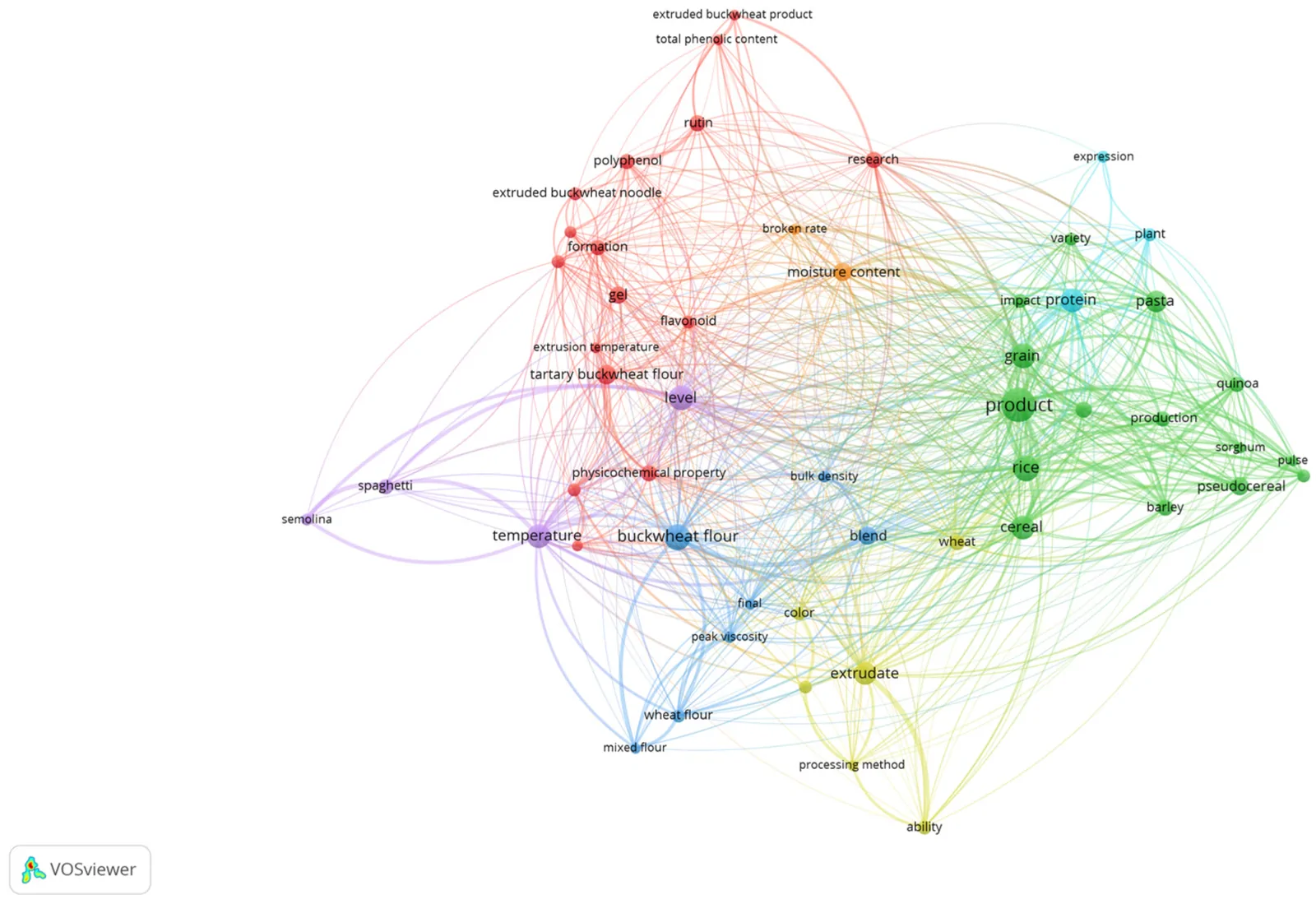
Keyword co-occurrence network map of the scientific literature on buckwheat in the context of extrusion and food processing, generated with VOSviewer based on a database query (2000–2026; n = 198, minimum co-occurrence threshold = 5).

The keyword network generated for buckwheat-related extrusion studies highlights strong associations among extrusion technology, starch characteristics, antioxidant activity, flavonoids, and product quality, Figure 3. Rutin-related terms remain prominent within the network, reflecting the importance of preserving bioactive compounds during processing. Optimisation and modelling keywords also appear among the identified clusters, indicating increasing application of quantitative approaches for evaluating process–response relationships in buckwheat-based systems.

Buckwheat exhibits extrusion behaviour that differs from quinoa and amaranth because of its starch structure and high flavonoid content. Several studies have reported that processing conditions influence expansion characteristics, density, and flavonoid retention, particularly rutin stability. Because nutritional quality and physical quality do not necessarily respond in the same direction to increasing process severity, buckwheat-based products represent a useful model system for multi-response optimisation approaches [47,48,49,50,51].

### 2.4. Blends and Composite Formulations

A comparative summary of the nutritional profiles of key pseudocereals and common cereals is given in Table 1. The technological and nutritional limitations inherent in extruding pseudocereal flours as sole ingredients have driven interest in composite and blend formulations. Pseudocereal–cereal blends, notably quinoa–corn, amaranth–wheat, and buckwheat–rice systems, maintain acceptable expansion (ratio 3.0–4.5) at pseudocereal inclusion levels of 15–40% while purely pseudocereal systems often struggle to achieve expansion ratios above 3.0 without carrier starch support [52,53].

Lentil–quinoa extruded snacks enriched with pumpkin powder have been optimized using a Box–Behnken experimental design and a stepwise-response surface method, and the results showed that the optimized product was achieved with 44.2% pumpkin flour, 22% feed moisture, and 172.1 rpm screw speed. The optimized lentil–quinoa snack was a valuable source of fibre (15%), protein (17.3%), and antioxidants (TPC = 15.28 mg GAE.g^−1^ and DPPH = 33.66%) [54]. Functional ingredient incorporation—including vegetable powders, dietary fibre concentrates, and omega-3 fatty acid sources—introduces additional complexity into the mathematical modelling challenge, requiring expanded multi-response optimisation frameworks [55,56,57,58].

**Table 1:** Summary of nutritional compositions of major pseudocereals compared to common cereals (per 100 g dry weight basis).

Parameter	Quinoa	Amaranth	Buckwheat	Wheat	Maize	Rice
Protein (% DW)	12–23	14–17	11–15	10–15	8–12	6–10
Fat (% DW)	6–8	6–9	2–4	1–2	3–6	1–3
Starch (% DW)	58–68	65–68	56–70	60–75	62–78	70–80
Dietary fibre (% DW)	7–10	10–15	10–16	10–15	7–12	2–4
Ash (% DW)	3–4	3–4	2–3	1–2	1–2	0.5–1.5
Lysine (g/100 g protein)	5.1–6.0	5.3–5.8	5.8–6.5	2.0–2.8	2.5–3.0	3.5–4.0
Gluten-free	Yes	Yes	Yes	No	Yes	Yes
Starch granule size (μm)	0.4–2.0	1–3	3–10	5–35	5–25	3–8
Gelatinisation temp (°C)	57–64	68–75	58–68	58–70	62–72	68–77
Key bioactives	Betacyanins, saponins, tocopherols	Rutin, quercetin, squalene	Rutin, quercetin, D-chiro-inositol	Lignans, ferulic acid	Zeaxanthin, carotenoids	γ-oryzanol, GABA

DW: dry weight basis. Data compiled from multiple literature sources [4,59,60,61,62,63,64].

The compositional differences summarized in Table 1 have important implications for mathematical modelling of extrusion processes. Compared with conventional cereals, pseudocereals generally contain higher protein, dietary fibre, minerals, and bioactive compounds, together with distinct starch characteristics such as smaller starch granules and different gelatinisation behaviour. These compositional differences substantially influence melt rheology, starch gelatinisation, bubble growth, and expansion during extrusion, resulting in more complex and highly nonlinear process–response relationships. Consequently, predictive models for pseudocereal extrusion must account for stronger interactions among formulation composition, moisture content, barrel temperature, and screw speed than models developed for conventional cereal systems. This complexity partly explains the increasing use of advanced modelling approaches, such as artificial neural networks and hybrid machine-learning techniques, which are better suited to capturing nonlinear relationships than conventional polynomial models. Therefore, the selection of an appropriate modelling approach depends not only on the extrusion process variables but also on the compositional characteristics of the pseudocereal matrix. Conventional polynomial models may adequately describe simple response surfaces, whereas highly nonlinear interactions associated with protein-rich and fibre-rich formulations often require machine-learning approaches with greater predictive flexibility.

## 3. Quality Attributes of Extruded Pseudocereal Snacks

### 3.1. Physical Properties

Physical properties remain the most frequently modelled responses in extrusion studies because they directly influence consumer perception and product quality. These quality attributes represent the principal response variables used in mathematical modelling studies and form the basis for model development, validation, and optimisation of extrusion processes. Expansion ratio (ER), bulk density (BD), texture, and colour are commonly used indicators of process performance and are routinely incorporated into optimisation frameworks. Among these responses, ER and BD are the most widely reported because they reflect the extent of structural development occurring during die expansion [65,66,67,68,69].

The definition of ER is the ratio of the cross-sectional diameter of the extrudate to that of the die opening. It is the single most widely modelled quality response in the extrusion literature. For extruded pseudocereal snacks, reported ER values range from 1.5 to 6.5, with optimal values for snack acceptance generally between 2.5 and 4.5 [65,66]. BD is inversely correlated with ER and usually ranges from 50 to 200 g/L for acceptably expanded pseudocereal extrudates [67]. Textural properties—including hardness, crispness, fracturability, and chewiness—are measured by texture profile analysis (TPA), with hardness values often ranging from 15 to 120 N, and consumer preference studies generally favouring the range of 20–55 N for crispy snack applications [68].

Colour attributes, quantified by the CIE L*, a*, b* colour space, serve as critical quality indicators. In extruded pseudocereal snacks, L* values normally range from 55 to 85, a* (redness) from −1 to +5, and b* (yellowness) from 12 to 30, with barrel temperature exerting the dominant effect on colour development through Maillard browning kinetics [69]. The internal microstructure of extrudates, characterized by SEM and X-ray microtomography, reveals mechanistic insights into the physical expansion process. Open-pore, thin-walled microstructures with uniform pore-size distributions (typical pore diameters of 0.5–3 mm) are associated with desirable crispy textures and high sensory ratings [70].

### 3.2. Functional and Pasting Properties

The water absorption index (WAI) and water solubility index (WSI) reflect the extent of starch gelatinisation and polymer degradation during extrusion. WAI ranges from 3.5 to 8.5 g/g in pseudocereal extrudates and increases with processing severity up to an optimum, beyond which starch fragmentation reduces gel-holding capacity [71]. WSI, ranging from 5 to 25% in extruded pseudocereal systems, reflects the degree of starch dextrinisation; higher WSI values indicate greater starch breakdown and are associated with improved digestibility [67].

RVA parameters are frequently used to evaluate structural modifications induced by extrusion and have been associated with texture, expansion behaviour, and functional properties of extruded products [72].

### 3.3. Nutritional Properties

Extrusion processing affects the nutritional quality of pseudocereal products through a combination of thermal and mechanical effects. Depending on processing conditions, extrusion may improve protein digestibility, reduce antinutritional factors, alter starch digestibility, and influence the stability of bioactive compounds [73,74]. Because pseudocereals are frequently promoted as functional ingredients, understanding and predicting these changes is important when optimizing extrusion conditions.

The thermal and mechanical stresses generated during extrusion modify both protein and starch structures. Heat and shear can unfold protein molecules, expose hydrophobic groups, and disrupt disulfide bonds, while simultaneously promoting starch gelatinisation and molecular degradation. Moderate processing conditions generally improve in vitro protein digestibility (IVPD) and reduce antinutritional factors such as trypsin inhibitors and phytates, which are commonly present in pseudocereals. Excessive processing intensity may promote Maillard reactions and protein cross-linking, reducing lysine availability and the retention of other heat-sensitive nutrients. Changes in starch digestibility are similarly affected by extrusion conditions and are therefore frequently included in predictive modelling and optimisation studies aimed at balancing nutritional and technological quality [72,73,74,75,76,77].

The retention of bioactive compounds during extrusion is an important consideration in pseudocereal product development because quinoa, amaranth, and buckwheat are recognized sources of polyphenols, flavonoids, squalene, and other health-promoting compounds. Numerous studies have demonstrated that extrusion conditions, particularly barrel temperature, feed moisture content, and specific mechanical energy, influence the stability of these compounds during processing [51,73,74,75,76,77].

### 3.4. Sensory Properties

Consumer acceptance of extruded pseudocereal snacks is strongly influenced by textural, visual, and flavour attributes. Sensory attributes are typically categorized into geometric–mechanical properties (texture, crispness, expansion) and metabolic properties (flavour, colour, Maillard compounds) [78,79].

The cross-sectional expansion ratio (SEI) and BD are the primary physical indicators of crispness and hardness. High-moisture feeds typically limit starch vapor expansion at the die exit, yielding dense, structurally rigid cellular walls that consumers find as hard or crunchy [65,66]. High temperatures reduce melt viscosity, encouraging rapid nucleation and bubble growth, which yields a highly expanded, fragile, crisp cell structure. Modelling these mechanical attributes involves connecting process inputs to instrumental crispness markers (such as peak force and acoustic emission counts) [68,70].

Colour alterations during extrusion are primarily governed by the overall thermal profile (Ea), which drives non-enzymatic Maillard browning between reducing sugars and amino acids. This reaction is trackable via the Hunter L*, a*, b* colour space system, where decreasing L* (lightness) and increasing a* (redness) values serve as direct kinetics responses for optimisation algorithms [23,32].

By mapping these sensory criteria alongside the nutritional and pasting attributes detailed above, a comprehensive multi-response framework is established.

## 4. Mathematical Modelling Approaches

The studies identified through the literature search were classified according to the mathematical modelling approach employed. The following sections therefore discuss each modelling framework separately, focusing on its theoretical basis, typical applications in pseudocereal extrusion, predictive performance, and practical limitations.

### 4.1. Response Surface Methodology (RSM)

RSM is one of the most commonly applied tools for studying and optimizing food processing operations involving several interacting variables. Since its introduction by Box and Wilson in the early 1950s, the method has been extensively used to evaluate the effects of process factors and to determine operating conditions that produce the desired product characteristics. Rather than attempting to describe the true relationship between variables and responses exactly, RSM uses relatively simple polynomial equations to represent process behaviour within the experimental range under investigation [54,80].

RSM remains the most frequently employed modelling approach in food extrusion research because it provides a practical framework for evaluating the effects of multiple processing variables simultaneously. In pseudocereal extrusion studies, RSM has been extensively used to investigate relationships between barrel temperature, screw speed, feed moisture content, and product quality responses such as expansion ratio, texture, colour, and nutritional properties [80,81,82,83].

#### 4.1.1. Experimental Designs Used in Pseudocereal Extrusion Studies

The central composite design (CCD), introduced by Box and Wilson (1951), is the most widely applied RSM design for pseudocereal extrusion optimisation. CCD comprises three types of design points: factorial points ($2^{ko}$ full or fractional factorial runs), axial (star) points positioned at distance α from the design center along each coordinate axis, and center point replicates for pure error estimation. The axial distance α is selected to confer rotatability ($\alpha =2^{k/4}$) or face-centring (α = 1), where *k* is the number of factors, the latter being preferred when axial points outside the factorial range are experimentally infeasible [84,85]. Face-centred central composite design (FCCCD) is particularly prevalent in food extrusion studies, as the constraint that all experimental points fall within the feasible range of process variables is critical for practical implementation [86].

The Box–Behnken design (BBD), introduced in 1960, is an alternative second-order response surface design that requires only three levels per factor and combines $2^{k-1o}$ incomplete block designs. BBD is notably more economical for three-factor studies (requiring 15 runs versus 20 for CCD with three centre points) and avoids extreme combinations of factor levels (i.e., all factors at their high or low levels simultaneously), which can be problematic for delicate food systems where combined extreme conditions might damage equipment or produce unacceptable products [87]. BBD has been extensively applied in amaranth and quinoa extrusion optimisation, where simultaneous high barrel temperature, high screw speed, and low moisture content could cause die plugging or burned extrudate [41,54,88].

Mixture designs constitute a specialized class of RSM designs applicable when the independent variables are constrained to sum to a constant (usually 1.0 or 100%) and are thus appropriate for optimizing blend compositions in pseudocereal–cereal–legume systems. Simplex–lattice and simplex–centroid mixture designs have been employed to optimize quinoa–amaranth–buckwheat ternary blends for gluten-free extruded product quality, simultaneously accounting for both formulation and process variable effects through combined mixture–process designs [89,90].

Standard RSM model development for pseudocereal extrusion follows a structured protocol: (1) identification of the critical independent variables and their operationally feasible ranges; (2) selection of the appropriate experimental design and determination of the required run number; (3) randomized execution of all experimental runs to control for systematic bias; (4) measurement of all selected response variables; (5) regression model fitting and statistical assessment; (6) verification of model assumptions; and (7) numerical optimisation, using the desirability function approach of Derringer and Suich (1980), followed by experimental confirmation of the predicted optimum [91,92]. Model validation through a set of independent confirmation runs distinct from the design matrix is an essential step that is often omitted in a substantial portion of the published RSM literature on food extrusion [81,87,93].

#### 4.1.2. Applications of RSM in Pseudocereal Extrusion Optimisation

Several studies have successfully applied RSM to optimize extrusion conditions for pseudocereal-based products, particularly focusing on barrel temperature, feed moisture, and screw speed as critical process variables [19,41,92,94,95,96]. Studies on amaranth- and quinoa-based extrudates have confirmed significant effects of extrusion conditions on expansion characteristics, WAI and WSI [97]. Valcárcel-Yamani and Lannes [97] comprehensively reviewed quinoa and amaranth applications in cereal-based foods and highlighted the increasing use of RSM in pseudocereal product optimisation.

For breakfast cereal development, Sukumar et al. [92] optimized a quinoa–finger millet–red rice gluten-free extruded breakfast cereal using RSM and a Box–Behnken design, achieving optimized processing conditions (barrel temperature 130 °C, screw speed 350 RPM, and feed moisture content 20 g/100 g) with a desirability of 0.937. Eftekhariyazdi et al. [54] validated that lentil–quinoa extruded snacks enriched with pumpkin responded to a four-factor RSM design with all second-order models showing the effect of process parameters (pumpkin flour ratio, barrel screw speed, and feed moisture content) on physical properties of lentil–quinoa–pumpkin extruded product. The optimal production conditions for process parameters were a pumpkin ratio of 44.2%, screw speed of 172.1 rpm, and feed moisture content of 22%.

Table 2 summarizes representative RSM experimental designs and their applications in pseudocereal extrusion optimisation.

#### 4.1.3. Advantages and Limitations of RSM

RSM offers several advantages for food extrusion optimisation including a well-established statistical theory with clear model interpretability and transparent quantification. The polynomial form of the model allows direct analytical solution for optimal conditions; relatively small experimental run numbers (15–30 for two to four factors) minimize cost and materials; the commercial software (Minitab, Design-Expert, SAS, R) is widely accessible; and the graphical visualisation tools (3D surface plots, contour plots, perturbation plots) facilitate intuitive understanding of factor–response relationships [100,101]. RSM is constrained to low-order polynomial approximations that are inadequate for capturing highly non-linear response behaviour; the assumption of a continuous, smooth response surface may not hold in extrusion systems where phase transitions (e.g., protein denaturation, starch gelatinisation) introduce abrupt discontinuities [100,102]. RSM is further limited by strict assumptions of normally distributed residuals, homoscedasticity, and absence of systematic model bias, and it allows no predictive information outside the experimental design region [103].

Overall, RSM remains the preferred approach for experimental optimisation involving a limited number of process variables, whereas its predictive capability decreases as process complexity and nonlinearity increase.

### 4.2. Artificial Neural Networks (ANNs)

ANNs are among the most frequently applied machine-learning tools for modelling food processing operations because they can describe nonlinear relationships between process variables and product responses without requiring predefined mathematical equations [104]. This characteristic is relevant for extrusion systems, where temperature, moisture content, screw speed, feed rate, and formulation variables interact in a complex manner [105].

Among the available ANN architectures, multilayer perceptron (MLP) networks trained using back-propagation algorithms are the most used in food extrusion studies. Typical ANN models employ extrusion process variables as inputs and predict physical, functional, nutritional, or sensory properties of the final product as outputs [106,107].

Compared with conventional regression-based approaches, ANN models are generally better suited to describing nonlinear behaviour and multiple interactions among variables, particularly when large datasets are available [103,108,109].

#### 4.2.1. Network Architectures, Training Algorithms and Model Validation

Most ANNs applications in food extrusion are based on feed-forward multilayer perceptron networks because of their flexibility and relatively simple implementation. These models have been successfully applied to predict expansion ratio, bulk density, water absorption index, water solubility index, texture, colour, and nutritional attributes using extrusion process parameters as predictors [77,110].

Alternative architectures, including radial basis function networks (RBFN), generalized regression neural networks (GRNN), and recurrent neural networks (RNN), have also been investigated [111]. Their application remains relatively limited compared with conventional MLP models. The choice of architecture is often influenced by dataset size, response complexity, and the intended application. For most laboratory-scale extrusion studies involving fewer than one hundred observations, simple MLP structures remain the preferred solution because they provide a balance between predictive performance and model complexity [112].

The Levenberg–Marquardt (LM) algorithm, a second-order optimisation method combining the advantages of gradient descent and Newton’s method, is the most widely employed in food extrusion modelling due to its rapid convergence characteristics for small-to-medium scale networks [109]. Bayesian regularisation (BR) training, which supplements the standard error minimisation objective with a regularisation term penalizing large network weights, effectively controls overfitting in small experimental datasets characteristic of food extrusion studies (often 15–50 data points) and generally produces networks with superior generalisation performance compared to LM-trained networks validated by early stopping [113]. Scaled conjugate gradient (SCG) and resilient back-propagation (Rprop) algorithms offer computational efficiency advantages for large networks at the cost of slightly reduced convergence speed [114].

Model validation commonly follows a three-partition scheme, for example 60/20/20% or 70/15/15% for training, validation, and test datasets, respectively. Performance metrics include coefficient of determination (R^2^), root mean square error (RMSE), mean bias error (MBE) and mean percentage error (MPE) [108]:

(1) $$RMSE={\left[\frac{1}{N}\cdot \sum_{i=1}^{N}{\left(x_{pre,i}-x_{\exp,i}\right)}^{2}\right]}^{1/2}$$

(2) $$MBE=\frac{1}{N}\cdot \sum_{i=1}^{N}\left(x_{pre,i}-x_{\exp,i}\right)$$

(3) $$MPE=\frac{100}{N}\cdot \sum_{i=1}^{N}\left(\frac{\left|x_{pre,i}-x_{\exp,i}\right|}{x_{\exp,i}}\right)$$

where *x_exp,i_* denotes the experimental values, *x_pre,i_* denotes the value predicted by the model, *i* denotes the observation index, and *N* is the total number of observations.

Although high predictive accuracy is frequently reported, model validation remains one of the major limitations of ANN-based extrusion studies. Many published models are developed using relatively small datasets and are evaluated using internal validation procedures only. Exceptionally high coefficients of determination should be interpreted with caution because model performance may decrease when applied to independent datasets or different extrusion systems. The development of larger datasets and external validation strategies remains an important challenge for future research.

The industrial applicability of many published machine-learning models remains uncertain because they have been developed using relatively small laboratory-scale datasets generated under controlled experimental conditions. Although such datasets are valuable for proof-of-concept studies, they often contain a limited number of observations and a narrow range of processing conditions, increasing the risk of model overfitting. Models that achieve excellent predictive performance during internal validation may not generalize well to independent datasets, different raw material batches, or industrial extrusion systems. Future research should therefore prioritize the development of larger and more diverse datasets, multi-site validation studies, and external validation under industrial processing conditions to improve model robustness and practical applicability.

#### 4.2.2. Applications in Pseudocereal/Cereal Extrusion Prediction

The application of ANN modelling to predict extrudate quality attributes from process variables has higher predictive accuracy compared to RSM polynomial models in the food extrusion literature, including pseudocereal systems [102,110]. Shihani et al. [110] reported that ANN models (R^2^ = 0.564–0.990) generally performed better than RSM models (R^2^ = 0.536–0.955) in predicting specific mechanical energy, expansion ratio, water absorption index, water solubility index, and sensory characteristics of the extruded flour blend.

Lončar et al. [115] used ANN modelling to predict and optimize the physical and technological properties of quinoa-enriched extruded products. The developed ANN model (MLP 7-6-5) proved to be effective, with a high coefficient of determination (r^2^ = 0.932). Filipović et al. [35] successfully optimized a corn-based extruded snack with quinoa flour to improve the nutritional, functional, and sensory quality while maintaining desirable technological properties using RSM to model the effects of quinoa addition and screw speed on 56 quality responses. In comparative studies, ANN models frequently outperform RSM. Huang et al. [116] used the back-propagation ANN (bp-ANN) to predict texture characteristics and antioxidant activities, where the optimized model structures had two hidden layers, one with ten neurons per layer (R^2^ ≥ 0.999) and another one with eight neurons per layer (R^2^ ≥ 0.993), while the corresponding linear fitting model achieved lower predictive performance (R^2^ = 0.913 and R^2^ =0.952), indicating the limitations of conventional linear models in capturing the complex nonlinear interactions inherent in extrusion systems. Thakur et al. [117] extruded preprocessed flours (malted yellow split pea, malted pearl millet, roasted pearled barley, and roasted quinoa) using a twin-screw extruder, and product characteristics were modelled as functions of die head temperature, screw speed, and feed moisture content using both ANN and RSM approaches. Hybrid optimisation techniques, including RSM-GA, RSM-SA, and ANN-GA, were also developed. Although both models established good predictive capability, RSM outperformed ANN in describing variations in product characteristics, possibly due to its ability to account for quadratic and interaction effects in extrusion processing conditions. Table 3 summarizes representative ANN and/or RSM applications in food extrusion modelling.

The studies summarized in Table 3 indicate that ANN models generally achieve higher predictive accuracy than RSM when strong nonlinear relationships exist among extrusion variables. The direct comparison among studies remains difficult because different raw materials, extrusion systems, response variables, and validation procedures have been employed. In addition, most published studies are based on laboratory-scale experiments involving relatively small datasets. The reported superiority of ANN models should be interpreted in the context of dataset size, model complexity, and validation strategy.

No single modelling approach is universally superior for all extrusion applications. RSM remains attractive because of its transparency, relatively low experimental requirements, and ease of interpretation. Its predictive performance may decrease when complex nonlinear interactions exist among process variables.

Artificial neural networks generally provide higher predictive accuracy for complex systems but require larger datasets and often lack interpretability. Hybrid approaches, including ANN-GA and ANFIS-based models, offer additional flexibility for multi-response optimisation and may combine predictive capability with improved decision support. Their application in pseudocereal extrusion remains relatively limited.

The model selection should be based not only on predictive performance but also on factors such as dataset size, intended application, interpretability requirements, computational complexity, and the availability of validation data.

### 4.3. Hybrid and Advanced Modelling Techniques

#### 4.3.1. ANN-Genetic Algorithm (ANN-GA) Optimisation

Hybrid ANN-genetic algorithm (ANN-GA) approaches combine the predictive capability of neural networks with the optimisation ability of evolutionary algorithms. In these systems, the ANN model is first developed to predict product responses, after which the genetic algorithm searches for processing conditions that maximize or minimize selected responses [123,124,125,126].

Compared with conventional desirability-based optimisation, ANN-GA approaches are better suited to exploring complex nonlinear response surfaces and simultaneously optimizing multiple quality attributes. Several studies have reported improved optimisation performance using ANN-GA frameworks, particularly when interactions among extrusion variables are difficult to describe using polynomial models [127,128,129,130]. The practical benefits of these approaches depend strongly on the quality and representativeness of the underlying ANN model.

#### 4.3.2. Adaptive Neuro-Fuzzy Inference Systems (ANFIS)

Adaptive neuro-fuzzy inference systems (ANFIS) combine neural-network learning with fuzzy-logic reasoning. In food process modelling, ANFIS offers the advantage of describing nonlinear relationships while simultaneously providing interpretable rule-based outputs [131,132,133,134].

Applications of ANFIS in food engineering have demonstrated predictive performance comparable to ANN models, frequently reporting coefficients of determination above 0.95 [135,136,137]. Despite these promising results, applications specifically focused on extrusion processing remain limited. Additional studies are required to determine whether the improved interpretability of ANFIS models provides practical advantages over conventional ANN approaches in pseudocereal extrusion systems.

#### 4.3.3. Support Vector Machines (SVM)

Support vector regression (SVR) has attracted attention as an alternative machine-learning method for modelling complex food-processing systems. One of its principal advantages is the ability to achieve good predictive performance using relatively small datasets, which are common in experimental food engineering research [138,139].

Although SVR has been successfully applied in several food quality prediction studies, its use in extrusion modelling remains relatively limited compared with RSM and ANN approaches. Available evidence suggests that SVR can provide predictive accuracy comparable to ANN models [140,141]. The number of extrusion-specific studies remains insufficient to establish clear performance advantages. Further comparative studies are needed, particularly for pseudocereal extrusion systems.

#### 4.3.4. Random Forest and Ensemble Methods

Ensemble learning approaches, including random forest (RF), gradient boosting machines (GBM), and extreme gradient boosting (XGBoost), have recently emerged as promising tools for food quality prediction [142,143,144,145]. These methods provide estimates of variable importance, allowing researchers to identify the processing factors most strongly associated with specific product attributes.

Applications in extrusion processing remain relatively limited but are increasing. Preliminary studies suggest that ensemble methods can achieve predictive performance comparable to ANN models while offering improved robustness against overfitting in some situations [140,146,147,148]. Their effectiveness in pseudocereal extrusion requires further validation using larger and more diverse datasets.

#### 4.3.5. Deep-Learning Approaches

Deep learning (including convolutional neural networks (CNN) and long short-term memory (LSTM) recurrent networks) has received increasing attention in food engineering because of its ability to model complex relationships and process large datasets. Applications include image-based quality assessment, process monitoring, and prediction of product properties [149,150,151,152].

Despite this potential, deep-learning applications in extrusion remain limited. Most published extrusion studies involve relatively small experimental datasets that are insufficient for effective training of deep neural networks [152]. Conventional ANN models continue to be the most widely used in the field. As larger datasets become available through industrial monitoring systems and digital manufacturing platforms, deep-learning approaches may become increasingly relevant for real-time process optimisation and control [23,153].

### 4.4. Mechanistic and Empirical Models

#### 4.4.1. Heat and Mass Transfer Models

Mechanistic modelling of food extrusion begins with governing equations for heat and mass transfer within the extruder barrel and die. The energy balance across a differential element of the extruder barrel integrates viscous heat dissipation (dominant at high screw speeds), heat conduction between the food melt and barrel wall, and the latent heat associated with phase transitions [20]. The specific mechanical energy (SME) in kJ/kg, defined as the mechanical energy input per unit mass of material processed, is a dimensionally meaningful mechanistic parameter that integrates the effects of screw speed, feed rate, and barrel geometry into a single scalar value correlating strongly with multiple quality responses:

(4) $$SME=\frac{2\pi \cdot N\cdot T}{Qmass}$$

where N is screw speed (rev s^−1^), T is measured torque (N·m), and Q_mass_ is the mass flow rate (kg s^−1^). SME has been demonstrated to correlate significantly (R^2^ 0.72–0.91) with WAI, WSI, bulk density, and expansion ratio in pseudocereal extrusion studies, serving as a mechanistically interpretable predictor that integrates multiple process variable effects [154,155,156,157].

#### 4.4.2. Residence Time Distribution (RTD) Modelling

Residence time distribution analysis characterizes the spread of residence times experienced by material elements traversing the extruder barrel, providing fundamental insights into mixing behaviour, product uniformity, and the intensity of thermal-mechanical treatment received. RTD in twin-screw food extruders is modelled as a combination of plug flow and continuous stirred tank reactor (CSTR) elements, with the relative contribution of each element depending on screw configuration, fill degree, and throughput [158,159]. Mean residence time (MRT) in twin-screw extruders processing pseudocereal flours ranges from approximately 45 to 120 s as screw speed increases from 100 to 300 rpm. RTD variance decreases with increasing screw speed, reflecting enhanced conveying efficiency and reduced axial back-mixing under more vigorous screw pumping action [160].

#### 4.4.3. Kinetic Models for Nutrient Degradation

The thermal degradation kinetics of heat-labile bioactive compounds in pseudocereals during extrusion are commonly described by first-order kinetic models of the Arrhenius type. For rutin degradation in buckwheat extrudates, first-order kinetic analysis across the temperature range 100–180 °C yields activation energies of 45–85 kJ/mol, enabling prediction of rutin retention as a function of time-temperature history within the extruder [43,161]. Combining heat transfer and kinetic modelling allows the construction of lethality-retention maps for bioactive compounds as a function of extrusion processing conditions, providing a mechanistically grounded basis for process optimisation targeting bioactive preservation [162,163,164].

From a model-selection perspective, the appropriate modelling framework should therefore be determined by the specific objective of the extrusion study rather than by predictive accuracy alone. RSM is particularly suitable for experimental optimisation and interpretation when the number of process variables and available observations is limited, whereas ANN and SVR become more appropriate when complex nonlinear process–response relationships dominate. ANFIS may be advantageous when nonlinear predictive capability needs to be combined with greater interpretability, while ANN-GA and related hybrid approaches are particularly useful for multi-response optimisation involving competing technological and nutritional objectives. Ensemble methods may provide an additional advantage when variable importance and predictive robustness are required. Thus, model selection in pseudocereal extrusion should represent a balance among dataset characteristics, response complexity, interpretability, validation requirements, and the intended predictive or optimisation objective.

## 5. Process Variables and Their Effects on Product Quality

### 5.1. Independent Variables in Extrusion Modelling

*Barrel Temperature*

Barrel temperature is generally considered one of the most influential processing variables in food extrusion because it affects starch gelatinisation, melt viscosity, expansion behaviour, colour development, and nutrient stability. Increasing temperature typically promotes starch transformation and expansion, although the magnitude of the response depends on feed composition and moisture content [162,163,164].

In pseudocereal-based systems, maximum expansion is frequently observed at intermediate-to-high barrel temperatures. Higher temperature increases may reduce melt viscosity excessively, resulting in structural collapse and deterioration of textural properties. Temperature also influences colour development through Maillard reactions and can affect the retention of thermally sensitive compounds, including polyphenols and flavonoids [165,166,167,168,169,170].

Mathematically, temperature effects on most extrusion quality attributes are more accurately captured using quadratic polynomial terms in RSM or nonlinear activation functions (e.g., sigmoid) in ANN models, as linear terms alone are often insufficient to describe the observed curvature and interaction effects reported in the literature [111,171].

*Screw Speed*

Screw speed influences both residence time and mechanical energy input during extrusion. In general, increasing screw speed increases shear forces and specific mechanical energy while reducing the time available for thermal treatment. However, the resulting effects on product quality depend strongly on feed composition and operating conditions, and consistent trends are not always observed across different studies [18,156]. For expansion ratio, screw speed generally shows a positive linear effect at sub-optimal temperatures and a positive-then-declining quadratic effect at optimal temperatures [172]. High moisture content attenuates the effect of screw speed on expansion ratio due to melt plasticisation, which reduces the sensitivity of starch gelatinisation to mechanical shear energy, leading to a significant xSS × xMC interaction in RSM models [162,173].

*Feed Moisture Content*

Feed moisture content is one of the most critical variables in pseudocereal extrusion, acting simultaneously as a plasticizer, a reactant in starch gelatinisation, a heat transfer medium, and a driver of steam-induced expansion at the die exit. Typical moisture levels for snack extrusion range from approximately 12–20% (wet basis). At lower moisture levels, increased melt viscosity leads to higher screw torque and SME input, generally promoting greater expansion, whereas higher moisture levels result in excessive plasticisation, reduced SME, and denser, less expanded, and more gummy extrudates [156,174]. The non-linear, inversely related response of WAI to moisture content (WAI increasing as moisture decreases, reflecting more extensive starch disruption at lower moisture) and the positively related response of WSI (reflecting greater dextrinisation at lower moisture) create characteristic crossed response surfaces well-suited to RSM polynomial modelling [154,175].

Although feed moisture content is consistently identified as a major extrusion variable, the direction and magnitude of its effects vary among studies. Differences in raw material composition, screw configuration, and barrel temperature frequently lead to contrasting responses, highlighting the importance of considering interactions among process variables rather than interpreting individual effects in isolation.

*Feed Rate and Composition*

Feed rate (or throughput) affects the fill degree of the extruder barrel, which in turn influences residence time, SME, and the energy balance within the system. Higher feed rates usually reduce SME input per unit mass (as the screw-to-barrel friction geometry changes), reduce mean residence time, and decrease the expansion ratio [18,117,174]. Feed composition, particularly the protein–starch–fat–fibre ratio, interacts with all process variables. Protein enrichment in pseudocereal blends (above 15% *w*/*w*) is generally associated with reduced expansion due to competition for water and disruption of the continuous starch matrix [159,176]. Fat content above approximately 4–5% acts as an internal lubricant reducing melt viscosity and expansion, whereas higher dietary fibre levels (5–8%) disrupt the continuity of the starch matrix, limiting bubble growth and increasing bulk density [156,157,159,177].

*Die Geometry and Diameter*

Die geometry and die diameter are less frequently varied in laboratory-scale optimisation studies but exert significant effects on extrudate shape, expansion anisotropy, and pressure drop across the die. Die diameter strongly influences the pressure drop-flow rate relationship in the die: smaller die diameters produce higher die pressures, more rapid flash evaporation of steam on die exit, and potentially greater radial expansion, but also higher specific mechanical energy input through increased back-pressure effects [18]. Mathematical models for die flow in pseudocereal extrusion employ power-law viscosity models to predict die pressure as a function of apparent shear rate, moisture content, and temperature, providing mechanistic inputs to SME calculation and expansion prediction [156,157,177].

### 5.2. Multiple Response Optimisation

The desirability function approach of Derringer and Suich (1980), universally implemented in commercial RSM software, converts each quality response to a dimensionless desirability value *d_i_* ∈ [0, 1] using individual desirability functions that reflect whether the response should be maximized, minimized, or targeted to a specific value with defined tolerance bounds [178]. The overall desirability D is then calculated as the geometric mean of individual desirability values:

(5) $$D={\left(\prod_{i=1}^{k}d_{i}^{w_{i}}\right)}^{1/\sum_{i=1}^{k}w_{i}}$$

where *d_i_* is the individual desirability of response *i*, *w_i_* is the corresponding importance weight, and *k* is the number of responses. The overall desirability D is maximized over the experimental design space to identify the optimum process conditions. In pseudocereal extrusion studies, simultaneous optimisation commonly involves maximizing expansion ratio, protein digestibility, and antioxidant activity while minimizing hardness, bulk density, and colour change (ΔE), with D values for reported optima normally ranging from 0.72 to 0.91 [100,178].

The major issue in pseudocereal extrusion is that processing conditions favouring physical quality are not always optimal for nutritional retention. Conditions that maximize expansion and crispness may simultaneously reduce the concentration of heat-sensitive bioactive compounds. Multi-response optimisation remains essential for balancing technological and nutritional objectives.

In multi-response extrusion optimisation problems, many quality attributes exhibit inherent trade-offs that cannot be simultaneously resolved to their individual optima. A fundamental trade-off in pseudocereal extrusion is the inverse relationship between expansion ratio (maximized at high temperature and low moisture) and bioactive compound retention (maximized at low temperature and high moisture), creating a Pareto frontier in the two-objective optimisation space [73,157,179]. Pareto optimisation, implemented through multi-objective evolutionary algorithms (MOEA) coupled with ANN surrogate models, provides decision-makers with the complete set of Pareto-optimal solutions rather than a single compromised optimum, enabling scientifically informed selection of operating conditions based on application-specific priorities [180].

## 6. Comparative Analysis of Modelling Approaches

To facilitate the comparative analysis presented in this section, Table 4 summarizes the rationale for selecting the principal mathematical modelling approaches, the types of extrusion variables and quality responses they are intended to analyse, and the information that can be obtained from their application. This overview provides a practical framework for selecting appropriate modelling strategies according to the characteristics of the extrusion system, the target quality attributes, and the intended modelling objective.

### 6.1. Prediction Accuracy and Model Performance

Published studies consistently demonstrate that advanced machine-learning approaches, particularly ANN-based and hybrid models, often achieve higher predictive accuracy than conventional RSM models when modelling complex nonlinear relation-ships in extrusion systems. Across the representative studies included in this review, reported R^2^ values generally range from 0.97–0.99 for ANN-GA and XGBoost models, 0.95–0.98 for ANN-MLP and ANFIS models, 0.93–0.97 for SVM, 0.93–0.96 for random forest, and 0.85–0.93 for RSM models [117,118,127,137,147,151,156,164,181,182,183,184,185,186,187,188,189,190,191,192,193,194]. RMSE values for expansion ratio prediction are 0.05–0.15 for ANN models versus 0.15–0.35 for RSM models, and MAPE values are 1.5–3.5% versus 4.5–8.0% respectively.

Nevertheless, direct quantitative comparison of model performance across independent studies should be interpreted with caution because reported statistical indicators are strongly influenced by dataset size, response variables, experimental design, preprocessing procedures, and validation strategy. Representative ranges of predictive performance reported in the literature are summarized in Table 5 to provide an overview of current modelling capabilities rather than a definitive ranking of modelling approaches.

The reported R^2^, RMSE, MAPE, and correlation coefficients illustrate the range of model performance reported in the literature rather than providing conclusive evidence of the superiority of one modelling approach over another. In particular, RMSE values cannot be directly compared across studies because they depend on the scale and units of the response variable, whereas R^2^ is influenced by the variability of the underlying dataset. These methodological differences should be considered when interpreting comparative model performance, as standardized benchmark datasets and independent external validation remain limited in the current literature.

The principal objective of comparing these modelling approaches is not to identify a universally superior method but to determine which modelling framework is most appropriate for a specific extrusion problem. Model selection depends on the complexity of the process, the amount of available experimental data, the required level of interpretability, and the intended application, ranging from laboratory optimisation to industrial process control.

### 6.2. Practical Considerations

RSM implementation requires only basic statistical knowledge and is supported by widely available commercial software (Design-Expert, Minitab, JMP) and open-source tools (R packages rsm, FrF2; Python packages pyDOE, statsmodels), making it accessible to food scientists without specialized computational expertise [195]. ANN modelling requires greater programming competence but is supported by Python (TensorFlow/Keras, PyTorch, scikit-learn) and MATLAB (Neural Network Toolbox) libraries that provide pre-built network architectures, training algorithms, and validation frameworks [196,197]. Hybrid ANN-GA implementations add evolutionary computation libraries (DEAP, pymoo for Python; GA Toolbox for MATLAB) to the required software stack and require careful parameter tuning of GA operators to ensure convergence reliability [198].

From an industrial perspective, model selection should also consider economic feasibility and the technical skills available within the processing plant. Although advanced machine-learning and hybrid frameworks may provide superior predictive performance, their implementation may require additional investment in sensors, data-acquisition infrastructure, software, computational resources, model maintenance, and personnel training. The adoption of Industry 4.0 technologies in the food sector is still constrained by implementation costs, organisational barriers, and gaps between existing workforce competencies and the digital skills required for increasingly automated production environments [139,199,200].

These constraints may be particularly relevant to small- and medium-scale processing facilities with limited financial and technical resources. Therefore, improvements in predictive accuracy should be evaluated against implementation and maintenance costs and the expected operational benefits, including reduced raw-material losses, lower energy consumption, improved process stability, and more consistent product quality. Rather than assuming an inherent economic advantage of more complex models, their return on investment should be assessed on a case-by-case basis according to production scale, existing digital infrastructure, implementation costs, and measurable operational benefits. In some industrial settings, simpler and more interpretable approaches such as RSM may consequently provide a more favourable balance between modelling performance, implementation complexity, and economic feasibility, whereas advanced data-driven models may be justified when sufficient production scale, infrastructure, and technical expertise are available [199,200].

### 6.3. Application-Specific Recommendations

For initial screening and exploratory investigation of a novel pseudocereal extrusion system with limited prior knowledge, a two-phase approach is recommended: first, a Plackett–Burman or fractional factorial screening design to identify the most influential variables (reducing a potential 6–8 factor problem to the 3–4 most critical), followed by a three-to-four factor RSM optimisation within the ranges established by screening [201]. For applications requiring high prediction accuracy across a broad processing range—such as continuous online quality monitoring, adaptive process control, or digital twin development—ANN or ANN-GA hybrid approaches are recommended when suitable datasets are available, particularly if historical process data from previous production campaigns can supplement purpose-designed experiments used to build the training dataset [117,136]. For industrial-scale real-time process control requiring automated decision-making within computational time constraints of seconds, lightweight MLP networks or SVR models offer the best balance of prediction accuracy and computational speed; tree-based ensemble methods and deep-learning models entail computational latency incompatible with real-time control applications operating at 10–100 Hz sampling rates [202,203,204].

### 6.4. Critical Comparison of Modelling Approaches for Pseudocereal Extrusion

The suitability of a modelling approach depends on the study objective, dataset size and characteristics, experimental design, and intended industrial application. Typical Box–Behnken and central composite designs require only 15–30 experimental runs, making RSM economically attractive for food process development. The resulting polynomial equations are readily interpretable and facilitate understanding of factor interactions while providing mechanistic insight into extrusion behaviour.

ANN models offer superior flexibility for describing highly nonlinear relationships commonly encountered during extrusion cooking, including starch gelatinisation, protein denaturation, melt rheology changes, and expansion phenomena. ANN performance depends strongly on the size, quality, and representativeness of the available datasets. Many food extrusion studies are based on fewer than 50 experimental observations, increasing the risk of overfitting and limiting model generalisation beyond the experimental domain. Consequently, high predictive accuracy, particularly exceptionally high R^2^ values, should be interpreted cautiously unless supported by independent validation datasets or cross-validation procedures. Also, the transferability of laboratory-developed ANN models to industrial extrusion systems remains insufficiently investigated.

Hybrid approaches such as ANN-GA and ANFIS attempt to combine the predictive accuracy of machine-learning algorithms with optimisation capability or model interpretability. ANFIS provides the additional advantage of generating linguistically interpretable fuzzy rules, whereas ANN-GA is particularly effective for multi-objective optimisation involving competing quality responses such as expansion ratio, texture, antioxidant retention, and energy consumption. However, these approaches require greater computational expertise and remain less frequently implemented in industrial food production environments.

Among recently emerging machine-learning techniques, random forest, gradient boosting machines, and XGBoost have demonstrated promising performance in food engineering applications. In contrast to conventional ANNs, these algorithms provide variable importance metrics that facilitate interpretation of process–response relationships while maintaining high predictive accuracy. Their ability to handle nonlinear interactions, missing values, and heterogeneous datasets makes them attractive candidates for future pseudocereal extrusion modelling, particularly as larger industrial datasets become available.

A direct comparison of modelling approaches across published studies remains challenging because of substantial differences in dataset size, response variables, validation procedures, experimental designs, and extrusion systems. Consequently, no single modelling approach can be considered universally superior. RSM remains the preferred tool for process understanding and experimental optimisation using limited datasets, whereas ANN and other advanced machine-learning approaches become increasingly advantageous as dataset size, process complexity, and predictive objectives increase. Future research should focus not only on improving predictive accuracy but also on developing larger and more diverse datasets, implementing independent external validation, and evaluating model performance under industrial processing conditions to enhance robustness, interpretability, and practical applicability.

The transfer of laboratory-developed models to industrial-scale extrusion systems also requires consideration of physical and operational constraints that are rarely represented in laboratory datasets. Scale-up may alter residence-time distribution, heat and mass transfer, specific mechanical energy, shear conditions, and melt flow behaviour, while interactions between the food melt and barrel or die surfaces contribute to pressure development and viscous dissipation [20,156,157,177]. At industrial scale, additional sources of variability may arise from progressive equipment wear and from transient operating regimes during start-up, shut-down, and transitions between production conditions. These effects are generally not represented in laboratory datasets developed under controlled steady-state conditions and may therefore reduce model transferability to manufacturing environments. Consequently, models intended for industrial implementation should be validated under representative large-scale operating conditions and should account, where possible, for equipment condition and transient process behaviour.

## 7. Recent Advances and Emerging Trends

### 7.1. Integration of Machine Learning and Artificial Intelligence

The emergence of process analytical technology (PAT) frameworks in food manufacturing, paralleling their established application in pharmaceutical processing, has created new opportunities for data-driven modelling of extrusion processes through continuous in-line and on-line sensor deployment. Near-infrared (NIR) spectroscopy, coupled with principal component analysis (PCA) and partial least squares regression (PLS), can now provide real-time monitoring of moisture content, starch gelatinisation degree, and protein denaturation extent within the extruder barrel at measurement frequencies of 0.1–1 Hz, generating time-series datasets orders of magnitude larger than conventional batch-by-batch quality measurements [205,206,207]. Multivariate statistical process control (MSPC) charts constructed from PCA scores of these high-frequency sensor data enable early detection of process deviations before they manifest in finished product quality failures, representing an impactful change from reactive to proactive quality management [208,209,210].

The digital twin paradigm—wherein a continuously updated virtual model of the physical extrusion process runs in parallel with real operation, ingesting real-time sensor data and providing predictive outputs and control recommendations—is perhaps the most significant near-future transformation in food extrusion technology [210,211]. A complete digital twin for pseudocereal extrusion would integrate mechanistic models for heat and mass transfer, melt rheology, and specific mechanical energy; ANN or physics-informed neural network (PINN) surrogate models for quality attribute prediction; digital sensor integration for real-time state estimation; and model predictive control (MPC) algorithms for proactive process optimisation [151,212,213]. Physics-informed neural networks (PINNs), which embed known physical laws (conservation equations, thermodynamic constraints) as soft constraints within the neural network training loss function, offer the strong benefits of combining the expressiveness of neural networks with the physical consistency and data efficiency of mechanistic models—a combination especially valuable in food extrusion where data are scarce and physics are partially understood [214,215].

### 7.2. Sustainability and Circular Economy

The utilisation of by-products and waste streams from pseudocereal processing—including quinoa saponin-containing washing water, amaranth defatted meal after oil extraction, and buckwheat hull and bran fractions—as value-added ingredients in extruded snack formulation is an active and commercially relevant research frontier [23,216,217]. Mathematical modelling of extrudate quality from waste stream-incorporating formulations faces the additional complexity of highly variable raw material composition (dependent on primary processing conditions), requiring robust, composition-adaptive ANN models trained on data spanning the full range of waste stream compositional variability [218,219]. Life cycle assessment (LCA) integration with process optimisation models enables simultaneous optimisation of product quality and environmental impact (greenhouse gas emissions, water consumption, energy demand), creating genuinely sustainable extrusion process designs that balance product performance with ecological responsibility [220,221]. Energy consumption modelling in twin-screw extrusion, based on SME calculation and direct energy metering, delivers the quantitative foundation for energy efficiency optimisation alongside quality optimisation, contributing to the industrial circular economy agenda [222].

### 7.3. Personalized Nutrition and Functional Foods

Growing interest in personalized nutrition, together with developments in genomics and food technology, is encouraging the design of extruded snacks that better match individual dietary requirements and health goals. Mathematical modelling approaches capable of predicting nutrient bioaccessibility and in vivo bioavailability from extrudate structural parameters, rather than simply total nutrient content, are essential for evidence-based personalized snack development [223]. In vitro digestion models (INFOGEST standardized protocol) coupled with ANN prediction of digestion kinetics from extrudate structural parameters (pore size, wall thickness, starch crystallinity) have been demonstrated to be feasible tools for predicting the glycaemic index contribution of extruded pseudocereal snacks without requiring expensive and time-consuming in vivo clinical studies [224,225]. Substantiation of health claims for functional extruded pseudocereal snacks under EU Regulation 1924/2006 and FDA qualified health claim frameworks requires quantitative demonstration of bioactive retention and bioaccessibility, further reinforcing the need for mathematically rigorous, validated modelling of nutrient behaviour during extrusion processing [226].

## 8. Challenges and Future Perspectives

### 8.1. Current Limitations

The most fundamental limitation constraining mathematical modelling advances in pseudocereal extrusion is the fragmented, unstandardized nature of the published experimental literature. Raw material characterisation protocols are inconsistent between research groups, with particle size distribution, protein solubility fraction analysis, and starch crystallinity measurement by X-ray diffraction all commonly omitted from published studies, precluding cross-study model training and validation [157,227]. The absence of publicly accessible, machine-readable databases of pseudocereal extrusion experimental data—compared to those available in genomics, materials science, and pharmaceutical manufacturing—is a critical structural gap that prevents the application of data-hungry deep-learning methods to this domain [228]. Standardisation of measurement protocols for critical quality attributes would improve cross-study data comparability and enable meta-analytical model development [229].

A common challenge in food extrusion modelling is balancing predictive accuracy with model interpretability. While advanced approaches such as artificial neural networks and ensemble models often achieve excellent prediction performance (R^2^ values above 0.97 in some studies), they generally provide limited information about the underlying physicochemical mechanisms governing product responses [230]. It can be difficult to explain why a particular prediction is obtained or to confidently apply the model to new raw materials, formulations, or processing conditions that differ from those used during model development [231]. The emerging field of explainable artificial intelligence (XAI), including SHAP (Shapley additive explanations) values, LIME (local interpretable model-agnostic explanations), and attention mechanisms in deep learning, offers promising tools for post hoc interpretation of complex model predictions in food process modelling contexts [232].

A systemic weakness of published RSM and ANN models for pseudocereal extrusion is their validation exclusively within the experimental design region using the same raw material batch, extruder equipment, and analytical methods used in model development.

This limitation is particularly important for pseudocereals because raw material characteristics may vary among cultivars, growing environments, and harvest years. Varietal and environmental effects have been reported for compositionally relevant characteristics of pseudocereals, including protein fractions in amaranth and rutin content in buckwheat [30,39,167]. Such variability may alter the physicochemical and functional properties of the raw material and consequently modify its behaviour during extrusion. Therefore, machine-learning models trained using a single cultivar, harvest year, or raw material lot may show reduced predictive performance when applied to material obtained in subsequent seasons or from different growing environments.

True external validation, where a model trained on one laboratory’s data is applied to predict outcomes at a different laboratory using different raw material lots and extruder equipment, has been attempted in only a small fraction of published studies, and the results show substantial prediction error increases (RMSE 2–4-fold larger) attributable to raw material variability, equipment-specific effects, and analyst-dependent measurement differences [92,110,233].

To improve model robustness and transferability, future studies should include raw materials representing multiple cultivars, harvest years, growing environments, and production lots and incorporate relevant compositional descriptors as model inputs. Periodic model recalibration or updating may also be required when systematic changes in raw-material characteristics occur between production seasons.

### 8.2. Future Research Directions

Progress in machine learning for extrusion research will depend not only on advances in modelling techniques but also on the availability of high-quality data. Establishing shared, openly accessible databases of pseudocereal extrusion experiments that follow FAIR (findable, accessible, interoperable, reusable) data principles would facilitate data reuse, improve model development, and encourage collaboration across research groups [234]. A minimum dataset standard for pseudocereal extrusion studies should include: complete raw material characterisation (proximate composition, protein fraction solubility, starch crystallinity, particle size distribution, water activity); complete process variable records (all barrel zone temperatures, screw speed, feed rate, die geometry, measured SME and torque); and standardized quality attribute measurements for expansion ratio (image analysis), bulk density (graduated cylinder), hardness and crispness (TPA and three-point bending), colour (spectrophotometric CIE L*a*b*), WAI, WSI, in vitro protein digestibility (pH-stat method), total polyphenol content (Folin–Ciocalteu), and DPPH radical scavenging activity [23].

The integration of mechanistic process understanding with data-driven machine learning through physics-informed neural networks (PINNs) and hybrid grey-box models is the most theoretically sound and practically promising direction for advancing pseudocereal extrusion modelling beyond current capabilities [235]. By incorporating energy balance equations, gelatinisation kinetics, and protein denaturation thermodynamics as physics-based constraints within the neural network training procedure, PINNs can achieve accurate predictions with fewer experimental observations than purely data-driven black-box networks, directly addressing the persistent data scarcity challenge in laboratory food extrusion research [236].

The implementation of IoT-enabled smart extrusion systems, in which continuous sensor streams from multiple in-line sensors (torque, pressure, melt temperature at multiple barrel zones, NIR spectroscopy) feed real-time data to continuously updated predictive models, is the practical pathway from academic modelling to industrial intelligent manufacturing [237]. Model predictive control (MPC) algorithms operating on ANN surrogate models can enable proactive, anticipatory adjustment of barrel temperature setpoints and screw speed in response to detected raw material variability, maintaining consistent product quality without the 20–45 min response lag characteristic of conventional feedback control in batch manufacturing contexts [238].

The integration of consumer hedonic data and preference mapping into mathematical optimisation frameworks is a largely unexplored opportunity to bridge the gap between product engineering optima (defined in terms of physical and chemical quality attributes) and genuine consumer acceptance optima (defined by hedonic response, purchase intent, and willingness to pay) [239]. Preference mapping approaches, including internal and external preference mapping using partial least squares and principal component regression to relate hedonic scores to instrumental quality descriptors, provide the statistical link required to incorporate consumer palatability directly as an optimisation criterion in ANN-GA and multi-objective evolutionary frameworks for pseudocereal snack development [240].

## 9. Conclusions

Pseudocereals represent nutritionally exceptional and commercially timely raw materials for the development of extruded snacks. They offer complete essential amino acid profiles, rich bioactive compound content, natural gluten-free status, and favourable starch characteristics. Due to the complex and nonlinear challenges associated with their extrusion processing, pseudocereal-based products require rigorous mathematical modelling approaches for systematic optimisation.

Mathematical modelling has become an important component of extrusion research because of the complex interactions among process variables and product responses. Among the available approaches, response surface methodology remains the most extensively used tool owing to its simplicity, transparency, and relatively low data requirements. Its ability to represent highly nonlinear relationships is limited.

Artificial neural networks generally provide superior predictive performance when sufficient experimental data are available and have consistently outperformed conventional polynomial models in many food extrusion studies. Hybrid approaches, including ANN-GA- and ANFIS-based models, show additional potential for multi-response optimisation, although their practical application remains constrained by limited datasets and insufficient external validation.

Current evidence indicates that the major challenge is no longer the development of new modelling algorithms but rather the availability of robust experimental datasets and standardized validation procedures.

Future research should focus on integrating predictive modelling with process monitoring technologies, digital manufacturing systems, and real-time control strategies. Such developments could improve process efficiency, product consistency, and nutritional quality while supporting the broader implementation of data-driven extrusion technologies.

## Figures and Tables

**Table 2:** Comparison of experimental designs for RSM applied in pseudocereal extrusion optimisation.

Design Type	Number of Factors	Runs Required	Factor Levels	Key Features	Application Example	Reference
Central Composite Design (CCD)—Rotatable	3	20	5 (-α, −1, 0, +1, +α)	Rotatable; allows quadratic fitting; axial points may exceed feasible range	Quinoa–corn snack expansion optimisation	[19]
Face-Centred CCD (FCCCD)	3	17	3 (−1, 0, +1)	All points within feasible range; slightly less efficient but very practical	Amaranth extrudate WAI/WSI modelling	[98]
Box–Behnken Design (BBD)	3	15	3 (−1, 0, +1)	Economical; no corner points; safe for delicate food systems	Amaranth flour multi-response optimisation	[41]
Box–Behnken Design (BBD)	4	29	3 (−1, 0, +1)	Increased runs vs. 3-factor; allows 4 process variable study	Buckwheat–rice snack optimisation	[47]
Simplex–Centroid Mixture Design	3 mixture components	7–10	Proportions (0–1)	For blend optimisation; sum = 1 constraint	Quinoa–amaranth–buckwheat blend	[99]
D-optimal Design	4–6	Variable	Flexible	Computer-generated; handles constraints and mixtures simultaneously	Pseudocereal–legume formulation optimisation	[54]

CCD: central composite design; BBD: Box–Behnken design; WAI: water absorption index; WSI: water solubility index.

**Table 3:** Summary of representative ANN and RSM applications in food extrusion modelling with performance metrics.

Food System	ANN Architecture (Input-Hidden-Output)	Training Algorithm	Inputs	Output Responses	R^2^ (ANN)	R^2^ (RSM)	Reference
Multigrain beverage premix malted yellow split pea, malted pearl millet, roasted pearled barley, and roasted quinoa using twin-screw extrusion	ANN with one hidden layer consisting of five neurons had largest correlation coefficient	Levenberg-Marquardt	Die head temperature (140–180 °C), screw speed (40–50 rpm), feed moisture content (15–25%)	Water absorption index, water solubility index, bulk density, total colour change, specific mechanical energy, hardness	0.714–0.948	0.862–0.989	[117]
Wheat flour and wheat–black soybean blend (95:5) extruded using a single-screw Brabender extruder	A three-layer feed-forward network (3-8-4) consisting of one input layer one output layer and one hidden layer and a slightly larger three-layer feed-forward network (4-14-4) .	Backpropagation ANN	Barrel temperature (120–140 °C), feed moisture content (18–20%, d.b.), screw speed (156–204 rpm)	Specific mechanical energy, water absorption index, water solubility index, expansion ratio, crispness, hardness, appearance, overall acceptability	0.564–0.999	0.536–0.914	[110]
Gluten-free extruded breakfast cereal formulated from quinoa, finger millet, and red rice	Not applicable (study used only RSM)	Not applicable	Barrel temperature, screw speed, feed moisture content	Expansion ratio, bulk density, water absorption index, water solubility index, hardness, sensory acceptability	Not applicable	0.86–0.98	[92]
Consumer-ready flakes produced from Amaranthus viridis pseudocereal, soymeal, and modified corn starch	Not applicable (study primarily employed RSM optimisation)	Not applicable	Feed composition variables (pseudocereal, soymeal, modified corn starch proportions) and extrusion conditions	Nutritional composition, bioactive properties, antioxidant activity, sensory characteristics	Not reported	0.898–0.999	[118]
Functional wheat-based extruded products in twin-screw extrusion	Various configuration (3-n-7)	Levenberg-Marquardt training algorithm	Extrusion process conditions (barrel temperature; feed moisture content and screw speed)	Physical (ER, BD, RR, WSI, hardness, colour) functional (antioxidant, acrylamide and lycopene) and pasting properties (IPV, HPV, CPV) as well as system parameters (SME, FR, torque, fresh extruded product moisture content and product temperature)	0.945–0.999	Not reported	[119]
Fortified rice kernels (FRK) produced by twin-screw extrusion using broken rice flour and micronutrient premix	Back-propagation network with two number of the hidden layer used to predict the responses with varying numbers of neurons (5, 10, 15 & 20) of the input layer 4-n-1-1	Levenberg-Marquardt	Die head temperature, screw speed, feed moisture content, feeder screw speed	System responses (torque, die pressure, mass temperature), physicochemical properties (WAI, WSI, density), cooking properties (cooking time, losses, water absorption ratio)	0.963–0.990	0.941–0.980	[120]
Raw banana and defatted soy composite extrudates (gluten-free extruded snacks)	4-n-1-1 A three-layered feed-forward back-proportion algorithm	Levenberg-Marquardt	Barrel temperature (60–80 °C), screw speed (200–300 rpm), feed moisture content (10–20%), defatted soy flour content (0–32%)	Expansion ratio, product density, water absorption index, water solubility index, hardness, colour, and other physicochemical properties	0.909–0.991	0.379–0.918	[121]
Betaine-enriched spelt flour-based extrudates	Multilayer perceptron model consisted of three layers (input, hidden, and output) with hyperbolic tangent function as the activation function was used.	Broyden–Fletcher–Goldfarb–Shanno backpropagation ANN	Feed moisture content, barrel temperature, screw speed, betaine level	Expansion ratio, bulk density, hardness, SME, colour, texture-related responses	0.929–0.980	0.922–0.954	[122]

T: barrel temperature; SS: screw speed; MC: feed moisture content; FR: feed rate; ER: expansion ratio; BD: bulk density; SME: specific mechanical energy; WAI: water absorption index; WSI: water solubility index; AOA: antioxidant activity. R^2^ values represent test set performance. Adapted from compilation of multiple Scopus-indexed studies.

**Table 4:** Relationships between extrusion process variables, quality attributes, and recommended modelling approaches for pseudocereal extrusion.

Process Variable	Key Quality Outcomes	Recommended Modelling Approach	Model Suitability
Barrel temperature	Expansion ratio, bulk density, colour, phenolic retention	RSM, ANN	Strong quadratic effect, ANN better captures nonlinear behaviour at wider operating ranges
Feed moisture content	Expansion ratio, bulk density, WAI, WSI	RSM, ANN	Major process factor with nonlinear interactions
Screw speed	Expansion ratio, hardness, texture, SME	RSM, ANN	Influences shear and mechanical energy
Formulation (pseudocereal level)	Expansion, texture, nutritional quality, bioactive retention	Mixture design, ANN	Simultaneous optimisation of formulation and process variables
Specific mechanical energy	Starch gelatinisation, digestibility, WAI, WSI	ANN, SVR	Integrates the combined effects of several processing variables
Multiple interacting variables	Multi-response optimisation	ANN, ANFIS, ANN-GA, NSGA-II	Suitable for highly nonlinear, multivariable systems

**Table 5:** Comparative performance of mathematical modelling approaches for extruded pseudocereal/cereal snack quality prediction.

Modelling Approach	Typical R^2^ Range	RMSE (Expansion Ratio)	MAPE (%)	Data Requirement	Computational Complexity	Interpretability	Optimisation Capability
RSM (CCD/BBD)	0.85–0.93	0.15–0.35	4.5–8.0	Low (15–30 runs)	Very low	High	Analytical/numerical
ANN (MLP-BP)	0.95–0.98	0.05–0.15	1.5–3.5	Medium (30–80 data points)	Moderate	Low-moderate	Requires coupling with optimizer (GA, PSO)
ANN-GA Hybrid	0.97–0.99	0.04–0.12	1.2–2.8	Medium (40–100 data points)	High	Low	Global optimisation; multi-objective
ANFIS	0.95–0.98	0.05–0.14	1.5–3.0	Medium (30–60 data points)	Moderate	Moderate (fuzzy rules)	Gradient-based after training
SVM (RBF kernel)	0.93–0.97	0.08–0.18	2.0–4.0	Low-medium (20–60 data points)	Moderate	Low	Requires coupling with optimizer
Random Forest	0.93–0.96	0.08–0.20	2.4–4.5	Medium (50–150 data points)	Moderate	Moderate (feature importance)	Variable importance-guided
XGBoost	0.97–0.99	0.04–0.12	1.5–2.5	Medium-high (50–200 data points)	Moderate-high	Moderate	Tree-based optimisation
Mechanistic (SME-based)	0.72–0.91	0.20–0.50	6.0–12.0	Low (requires engineering data)	High (physics-based)	Very high	Physics-constrained

R^2^, RMSE, and MAPE values are ranges compiled from representative Scopus-indexed studies included in this review. Performance varies with specific food system, dataset size, and quality attribute. CCD: central composite design; BBD: Box–Behnken design; MLP-BP: multi-layer perceptron back-propagation; GA: genetic algorithm; ANFIS: adaptive neuro-fuzzy inference system; SVM: support vector machine; RBF: radial basis function; RF: random forest.

## Data Availability

No new data were created or analysed in this study.
